# Phase I Trial of Carboplatin and Gemcitabine Chemotherapy and Stereotactic Ablative Radiosurgery for the Palliative Treatment of Persistent or Recurrent Gynecologic Cancer

**DOI:** 10.3389/fonc.2015.00126

**Published:** 2015-06-05

**Authors:** Charles A. Kunos, Tracy M. Sherertz, Mazen Mislmani, Rodney J. Ellis, Simon S. Lo, Steven E. Waggoner, Kristine M. Zanotti, Karin Herrmann, Robert L. Debernardo

**Affiliations:** ^1^Department of Radiation Oncology, Summa Cancer Institute, Summa Health System, Akron, OH, USA; ^2^Department of Radiation Oncology, Case Comprehensive Cancer Center, University Hospitals Case Medical Center, Case Western Reserve University School of Medicine, Cleveland, OH, USA; ^3^Division of Gynecologic Oncology, Department of Obstetrics and Gynecology, Case Comprehensive Cancer Center, University Hospitals Case Medical Center, Case Western Reserve University School of Medicine, Cleveland, OH, USA; ^4^Department of Radiology, Case Comprehensive Cancer Center, University Hospitals Case Medical Center, Case Western Reserve University School of Medicine, Cleveland, OH, USA; ^5^Division of Gynecologic Oncology, Department of Obstetrics and Gynecology, Case Comprehensive Cancer Center, Cleveland Clinic, Cleveland, OH, USA

**Keywords:** stereotactic radiosurgery, radiation, carboplatin, gemcitabine, ovarian cancer, endometrial cancer, cervix cancer

## Abstract

**Background:**

We conducted a phase I trial to determine the safety of systemic chemotherapy prior to abdominopelvic robotic stereotactic ablative radiotherapy (SABR) in women with persistent or recurrent gynecologic cancers.

**Methods:**

Patients were assigned to dose-finding cohorts of day 1 carboplatin (AUC 2 or 4) and gemcitabine (600 or 800 mg/m^2^) followed by day 2 to day 4 Cyberknife SABR (8 Gy × three consecutive daily doses). Toxicities were graded prospectively by common terminology criteria for adverse events, version 4.0. SABR target and best overall treatment responses were recorded according to response evaluation criteria in solid tumors, version 1.1.

**Findings:**

The maximum tolerated dose of chemotherapy preceding SABR was carboplatin AUC 4 and gemcitabine 600 mg/m^2^. One patient experienced manageable, dose-limiting grade 4 neutropenia, grade 4 hypokalemia, and grade 3 nausea attributed to study treatment. One patient had a late grade 3 rectovaginal fistula 16 months after trial therapy. Among 28 SABR targets, 22 (79%) showed a partial response and 6 (21%) remained stable.

**Interpretation:**

Systemic chemotherapy may be given safely prior to abdominopelvic robotic SABR with further investigation warranted.

## Introduction

Ovarian, uterine, cervix, and vulvar cancers that recur or persist after initial treatment pose therapeutic management challenges. As many as 4 of every 10 women with recurrent or persistent gynecologic cancers have disease sites that abut organs that may have been previously taxed by chemotherapy or radiation, narrowly limiting clinically beneficial treatment options (1–3). In an effort to work around limits imposed by any prior treatment-related morbidity, investigators have explored whether stereotactic ablative radiotherapy (SABR) can be used safely and effectively as a non-invasive radiation treatment for women with abdominopelvic sites of recurrent or persistent gynecologic cancers (1–14). One phase II clinical trial indicated that a SABR-targeted gynecologic cancer disease control rate could be as high as 96% (1). But, in that same trial, it was noted that 62% of the SABR-treated patients had eventual non-SABR-targeted elsewhere disease progression (1). Whether a safe approach incorporating SABR and systemic chemotherapy could address both local and regional/distant gynecologic cancer disease has not been explored until now.

The rationale for this clinical trial was twofold. This first-ever SABR radiochemotherapy phase I trial evaluated safety concerns for the concurrent back-to-back administration of chemotherapy and high-dose radiation. By building upon an already characterized SABR dose of 24 Gy delivered in three consecutive daily doses of 8 Gy (1), we evaluated dose-escalated single intravenous administrations of carboplatin and gemcitabine chemotherapy administered before SABR for the purpose of early toxicity assessment of the back-to-back therapy. SABR was delivered by a robot-mounted linear accelerator [Cyberknife^®^, Accuray (Sunnyvale, CA, USA)] that enabled real-time cancer-target motion management and submillimeter radiation target accuracy. Carboplatin was selected for its DNA-damaging cytotoxicity and its relatively low-adverse event profile when compared to cisplatin (15, 16). Gemcitabine was selected for its inhibition of ribonucleotide reductase (RNRi) and its modest single-agent adverse event rate (17, 18). The second trial rationale addressed a clinical need for improved non-SABR targeted elsewhere disease control. Platinum-RNRi agent doublets range in activity from 16% in platinum-resistant recurrent ovarian cancer (19) to 50% in recurrent endometrial cancer (20). By studying back-to-back administration of chemotherapy and high-dose radiation, investigators had the opportunity to study clinical impact upon near-term tolerance of post-trial chemotherapy. Altogether, our objective was to identify a safe dose of carboplatin–gemcitabine chemotherapy preceding SABR – establishing a proof-in-concept that systemic chemotherapy may be administered safely before an ablative radiation course.

## Materials and Methods

This phase I single-center trial (NCT01652794) enrolled 12 patients between June, 2012, and March, 2014 to a regimen of dose-escalated carboplatin and gemcitabine chemotherapy given 1 day prior to 3 days of abdominopelvic robotic SABR for the treatment of recurrent or persistent gynecologic cancers (Table 1).

**Table 1:** **Patient and tumor characteristics (*n* = 12)**.

Characteristic	No. of patients	%^a^
**Age group**		
40–49	1	8
50–59	3	25
60–69	7	58
70–79	1	8
**Race**		
White	9	75
Black or African-American	3	25
**Ethnicity**		
Hispanic	0	0
Non-Hispanic	12	100
**Ecog performance status^b^**		
0	7	58
1	5	42
**Histology**		
Primary peritoneal cancer	1	8
Ovarian cancer	7	58
Uterine cancer	4	33
**Number of radiosurgery targets**		
1	3	25
2	3	25
3	5	42
4	1	8
**Radiosurgery targets**		
Abdominal lymph node	7	58
Liver	3	25
Vagina	2	17
**Pretherapy sum longest dimension**		
0–5 cm	4	33
5.1–10 cm	3	25
10.1–15 cm	3	25
15.1–20 cm	2	17

*^a^May not total 100 due to rounding*.

*^b^The Eastern Cooperative Group (ECOG) performance status reflects individual daily living activities on a scale of 0 (fully active with symptoms) to 5 (dead)*.

### Patients

All enrolled patients provided written informed consent and fulfilled the following criteria were 18 years or older, had one and up to four measurable sites of disease as defined by Response Evaluation Criteria in Solid Tumors (RECIST) version 1.1, had disease sites that had not undergone prior cryosurgery, radiofrequency ablation, or radiosurgery (although they may have had radiosurgery to another non-target disease site), had a performance status of 0 or 1, and had no severe congestive heart failure, angina, cardiac arrhythmia, uncontrolled hypertension, dyspnea at rest, renal function impairment (i.e., creatinine >2.0), or psychiatric illness. Patients also must have had at least one systemic chemotherapy regimen directed at recurrent or persistent gynecologic cancer, must be recovered from systemic chemotherapy for more than 28 days, and must have had any prior chemotherapy treatment-related toxicities resolve to less than or equal to grade 1. Patients must have had adequate organ function including absolute neutrophil count >1,500/mcl, platelets >100,000/mcl, hemoglobin ≥10 mg/dl, creatinine ≤2.0 mg/dl, bilirubin ≤1.5 × upper limit of normal (ULN), and aspartate aminotransferase, alanine aminotransferase, and alkaline phosphatase ≤2.5 × ULN. Exclusion criteria included pregnant women, patients with known anaphylaxis to carboplatin or to gemcitabine chemotherapy, patients with active lupus, dermatomyositis, Crohn’s disease, or ulcerative colitis, patients with human immunodeficiency virus actively taking antiretroviral therapy, patients with transplanted organs at-risk for lethal dysfunction or infection, patients with active non-gynecologic invasive malignancy (except treated non-melanoma skin cancer) within the previous 2 years, and patients with any history or evidence of active central nervous system disease [i.e., primary brain tumor, uncontrolled seizures, brain metastases, or cerebrovascular accident (stroke), transient ischemic attack (TIA), or subarachnoid hemorrhage]. University Hospitals of Cleveland and Case Western Reserve University (Cleveland, OH, USA) Institutional Review Board approval was granted for this phase I trial. The Case Comprehensive Cancer Center Data Safety and Toxicity Committee of University Hospitals of Cleveland and Case Western Reserve University provided oversight for this trial.

### Protocol treatment

This was a dose-finding phase I study of carboplatin and gemcitabine chemotherapy in combination with abdominopelvic robotic SABR. Carboplatin was obtained commercially and was administered as a 30-min continuous infusion for desired exposure (AUC) as determined by the Calvert formula (21). Gemcitabine was obtained commercially and was administered as a 30-min continuous infusion. A Fibonacci 3 + 3 cohort trial design was implemented for carboplatin–gemcitabine dose-escalation levels of AUC 2–600 mg/m^2^, AUC 4–600 mg/m^2^, and AUC 4–800 mg/m^2^, respectively. A single observed dose-limiting toxicity (DLT) event would lead to an additional three patients being treated at the dose level where the DLT occurred. Dose-finding escalation would continue if no additional DTLs were observed. Two observed DLTs would stop dose escalation, with the prior dose level being declared the maximum tolerated dose as long as six patients had been treated with less than one instance of DLT.

Stereotactic ablative radiotherapy involved three consecutive daily fractions of 8 Gy/fraction totaling 24 Gy using the robotic Cyberknife radiosurgery platform (Accuray). The robot arm-mounted linear accelerator delivered 6 MV radiation beams collimated by a tungsten-copper alloy iris aperture (1, 6). Gold fiducials or bony landmarks were used for image guidance during SABR dose delivery. SABR planning involved same-day thorax to mid-thigh non-contrasted contiguous axial 2-[^18^F] fluoro-2-deoxy-d-glucose positron emission tomography scans (FDG PET) and axial computed tomography or magnetic resonance imaging scans acquired in the head-first supine position following institutional protocol (22). FDG PET images were processed and co-registered for inverse radiation treatment planning using the MultiPlan 3.5.2 treatment planning system (Accuray). The clinical target volume (CTV) included the gross gynecologic cancer tumor volume (GTV) and any associated FDG PET signal extending around the GTV [i.e., thresholded 40% maximum target standard uptake value (22)]. A 3-mm margin was added to the CTV for a planning tumor volume (PTV). No more than four intended SABR targets could be treated on trial. An individual SABR target lesion volume could not exceed 160 cm^3^. Normal tissue contours were applied following convention – a peer-reviewed, video-complemented method for robotic SABR offers further specifics (6). Table 2 lists SABR radiation dose constraints.

**Table 2:** **Critical organ radiation dose constraints table**.

Organ	Total dose	Constraint 1	Total dose	Constraint 2	αβ ratio
Spinal cord	95%	<18 Gy	Not more than 0.3 cc	>20 Gy	2.5
Liver	67%	<17 Gy	Not more than 700 cc	<15 Gy	2.5
Heart	95%	<15 Gy	60%	<15 Gy	3.0
Partial right lung	95%	<12 Gy	60%	<8 Gy	4.0
Partial left lung	95%	<12 Gy	60%	<8 Gy	4.0
Both lung	95%	<12 Gy	60%	<8 Gy	4.0
Partial right kidney	90%	<14 Gy	50%	<10 Gy	2.4
Partial left kidney	90%	<14 Gy	50%	<10 Gy	2.4
Both kidney	90%	<14 Gy	50%	<10 Gy	2.4
Bladder	90%	<24 Gy	60%	<12 Gy	7.8
Rectum	90%	<24 Gy	60%	<10 Gy	3.0
Small bowel^a^	Not more than 1 cc	>24 Gy	N/A	N/A	8.4
Right femoral head	90%	<20 Gy	50%	<15 Gy	3.0
Left femoral head	90%	<20 Gy	50%	<15 Gy	3.0
Skin	95%	<24 Gy	50%	<12 Gy	10.5

*^a^Includes stomach*.

*cc, cubic centimeters; N/A, not applicable*.

### Safety assessments and follow-up

Patients had physical examinations and baseline hematological, hepatic, and renal function blood tests, baseline adverse event assessments [National Cancer Institute Common Terminology Criteria for Adverse Events (CTCAE), version 4.0], and baseline FDG PET scans within 35 days before the start of treatment. Hematological, hepatic, and renal function blood tests were repeated on days 8, 22, and 42. Platelet level and complete blood counts with differential were repeated on day 15. Posttherapy physical examinations and CTCAE adverse event assessments were repeated on day 42. Posttherapy FDG PET scans were mandatory and obtained on day 42 ± 5 days. Patients were considered “off-trial” after day 42, but patients were followed generally every 3 months thereafter by one of the treating physicians. Table 3 identifies protocol-defined adverse events occurring during therapy or within the first 6 weeks posttherapy.

**Table 3:** **Adverse events by grade with any relationship to protocol treatment (*n* = 12)**.

Toxicity	Grade				
	1	2	3	4	5
	No.	No.	No.	No.	No.
Blood/anemia	4	2	0	0	0
Constitutional (general)	0	0	0	0	0
Administration site pain	0	1	0	0	0
Anaphylaxis	0	0	1	0	0
Fatigue	9	3	0	0	0
Dermatology/skin	2	0	0	0	0
Gastrointestinal (general)	0	0	0	0	0
Abdominal pain	1	0	0	0	0
Constipation	2	0	0	0	0
Diarrhea	4	2	0	0	0
Dyspepsia	0	1	0	0	0
Emesis	2	0	0	0	0
Nausea	8	1	1	0	0
Infection (any site)	1	2	0	0	0
Investigations (general)	1	0	0	0	0
Alkaline phosphatase increased	2	0	0	0	0
Aspartate aminotransferase increased	2	0	0	0	0
Neutrophil count decreased	6	3	1	1	0
Platelet count decreased	12	2	1	0	0
White blood cell decreased	11	4	2	0	0
Metabolic/nutrition (general)	0	0	0	0	0
Anorexia	1	0	0	0	0
Dehydration	0	1	0	0	0
Hyperglycemia	1	0	0	0	0
Hyperkalemia	1	0	2	0	0
Hypoalbuminemia	3	1	0	0	0
Hypocalcemia	1	1	0	0	0
Hypoglycemia	1	0	0	0	0
Hypokalemia	1	0	2	1	0
Hypomagnesemia	2	0	0	0	0
Neurology	4	0	0	0	0
Pulmonary	1	0	0	0	0
Psychiatric (depression/insomnia)	1	0	0	0	0
Renal/genitourinary	0	0	0	0	0
Sexual/reproductive function	1	0	0	0	0
Totals	85	24	10	2	0

### Evaluation of clinical activity and statistical methods

Stereotactic ablative radiotherapy target responses were recorded following RECIST (23). SABR target FDG PET metabolic responses were assessed using previously outlined criteria (24, 25). Briefly, a complete metabolic response was absence of abnormal SABR target FDG uptake above cardiac blood pool FDG uptake. A partial metabolic response was a 15% or more reduction in abnormal SABR target FDG uptake. Stable metabolic response was recorded when there was a 25% or less increase or <15% reduction in SABR target FDG uptake. Progressive metabolic disease response was defined as a >25% increase in SABR target FDG uptake. Local disease relapse was recorded as disease progression of in-field SABR target(s). Elsewhere distant disease relapse was scored as any progression of disease out-of-field from SABR target(s). Time at-risk for disease progression or death was measured from the first date of trial carboplatin–gemcitabine chemotherapy until the date of the event. Descriptive and graphical statistics were computed using statistical software (SPSS 18.0, SPSS Inc., Chicago, IL, USA).

## Results

### Patients

Twelve patients underwent dose-escalated carboplatin–gemcitabine chemotherapy prior to abdominopelvic robotic SABR for the treatment of recurrent or persistent gynecologic cancers (Tables 1 and 2). All 12 (100%) received their prescribed carboplatin and gemcitabine infusions, all 3 prescribed SABR radiation treatments, and all are included in the treatment safety analysis. As of the date of data cutoff (March 12, 2015), all patients have been followed for >6-week on-trial period. The median follow-up is 21 months (range, 5–31 months).

Patients with recurrent or persistent ovarian (58%), uterine (33%), or primary peritoneal (8%) cancers were enrolled on this trial (Table 1). All patients had received prior chemotherapy for recurrent or persistent disease before carboplatin–gemcitabine–SABR treatment. Prior to trial enrollment, five (42%) had had prior conventional pelvic radiation and two (17%) had SABR to elsewhere sites of disease. On this trial, three patients received SABR within their prior conventional pelvic radiation fields. Patient pretherapy SABR target parameters are listed in Table 1. SABR treatment targeted lymph node sites of disease (including para-aortic, pelvic, or groin nodes) in 7 (58%) of the 12 patients. The median SABR target size (i.e., sum volume of all SABR targets up to four lesions, no individual lesion >160 cm^3^) was 72 cm^3^ (range, 7–248 cm^3^). Nine (75%) patients received adjuvant therapy following SABR, including chemotherapy or hormonal therapy starting after the 6-week trial period.

### Safety and tolerability

Twelve patients received 12 (100%) planned intravenous doses of carboplatin and gemcitabine chemotherapy on day 1 prior to day 2 to day 4 SABR treatments. Both 30-min infusions were well tolerated at all drug dose-escalation levels, with a single reversible hypersensitivity reaction to infusion occurring on day 1 in one patient. This patient was aggressively supported, medicated, and re-challenged such that both chemotherapy infusions were administered without subsequent incident.

Adverse events attributed to carboplatin–gemcitabine–SABR treatment are listed in Table 3. Most (97%) carboplatin–gemcitabine drug-related adverse events were mild to moderate in intensity (i.e., grade ≤3, resolving to grade 0–2 within 2 days). Two grade 4 dose-limiting toxicities, one each of hypokalemia and neutropenia, occurred in a single patient enrolled to the carboplatin AUC 4 and gemcitabine 600 mg/m^2^ dose level. This one patient was hospitalized on day 8 for these toxicities. The patient improved with supportive care and recovered to baseline function 1 day later. A total of five other patients at the carboplatin AUC 4 and gemcitabine 600 mg/m^2^ dose level had no dose-limiting toxicities. After dose escalation to carboplatin AUC 4 and gemcitabine 800 mg/m^2^, one diabetic patient had a single reversible grade 3 hyperglycemia event possibly related to treatments. Another patient at this dose level had a single reversible grade 3 neutropenia event possibly related to treatments. The phase I data safety and monitoring committee elected to stop carboplatin–gemcitabine dose administration at the AUC 4–800 mg/m^2^ level after three patients had been treated, and declared the AUC 4–600 mg/m^2^ level the maximum tolerated dose since six patients had been treated at that dose level with only a single DLT being observed. One patient, who was treated at the carboplatin AUC 4 and gemcitabine 600 mg/m^2^ level along with SABR directed at a rectovaginal recurrence (pretreatment volume 80 cm^3^) and who had prior surgery but no prior radiation therapy, developed a possibly treatment-related late rectovaginal fistula 16 months after trial therapy requiring a diverting colostomy. No carboplatin–gemcitabine–SABR treatment-related deaths occurred.

### Clinical activity

Among the 12 patients, carboplatin–gemcitabine–SABR controlled all targeted disease at 6 weeks posttherapy, with 22 (79%) targets labeled as partial responses and 6 (21%) targets labeled stable responses. A 6-week metabolic partial response (i.e., >15% decrease in target FDG SUV_max_) was achieved in 8 (67%) patients. The median decrease in FDG SUV_max_ was 39% (range, +6 to −76%). No local in-field SABR target disease progression has been recorded in the 6-week trial period. Disease progression outside of the SABR field occurred in nine (75%) patients, six of whom had received additional hormonal therapy or chemotherapy after the 6-week carboplatin–gemcitabine–SABR trial period. Four (33%) patients had a disease progression date more than 6 months after the start of carboplatin–gemcitabine–SABR treatment. Three of these four patients received additional post-trial chemotherapy. One patient with recurrent ovarian cancer has had a progression-free interval of 27 months. Two patients with recurrent uterine cancer have had progression-free intervals of 16 and 27 months.

## Discussion

Most gynecologic cancers are sensitive to radiochemotherapy with initial response rates exceeding 80% following surgery, chemotherapy, radiation, or a combination of these treatments. However, recurrent or persistent disease after initial anticancer intervention remains a substantial hurdle in the long-term control of these diseases. In women with recurrent or persistent gynecologic cancers, SABR has yielded good local control and a well-tolerated toxicity profile (1). But one major drawback of a SABR therapeutic strategy is its inability to target disease not discernable on diagnostic imaging. This phase I trial determined the maximum tolerated doses of carboplatin to be an AUC of 4 and gemcitabine to be 600 mg/m^2^ prior to three-fraction daily 8 Gy SABR treatments in a heavily pretreated population of patients. Although our selection of initial single-administration carboplatin–gemcitabine dose levels appeared to be conservative, hematological, and electrolyte toxicity did not allow for full dose escalation. Toxicity emerged typically by the second week after treatment, with one patient requiring hospitalization on day 8. Carboplatin AUC 4 and gemcitabine 600 mg/m^2^ doses were administered safely, but treatment was still associated commonly with hematological and electrolyte abnormalities in the 6-week posttherapy observation period. A longer observation period for late posttherapy toxicities would strengthen this study.

The concept of systemic and radiosensitizing chemotherapy administered prior to SABR for the treatment of recurrent or persistent gynecologic cancer does not have precedent. In this trial, carboplatin and gemcitabine were selected purposefully for their known clinical anticancer activity and their known tolerable safety profile. While it is difficult to determine whether anticipated systemic and radiobiological effects occurred in SABR and occult non-SABR targets, it is encouraging to find that carboplatin–gemcitabine–SABR did result in four (33%) patients having a disease progression date of more than 6 months posttherapy. But, three of these patients received at least one additional cycle of chemotherapy, and thus the results cannot be attributed to trial therapy alone. It is important to point out that our study was not designed to have sufficient power to comment upon important outcome differences among the enrolled patients. In one of our exploratory analyses, we found that eight (67%) patients achieved a metabolic partial response (i.e., >−15% reduction in SUV). Nevertheless, the greatest absolute reductions in FDG standard uptake value did not match the longest disease-free intervals. A more rigorous study of tumor FDG uptake heterogeneity and kinetics, a more homogenous patient cohort, and more uniform adjuvant management after the 6-week trial period would have strengthened our study.

There are exciting opportunities for implementing combined systemic chemotherapy and SABR in women with recurrent or persistent gynecologic cancers. Clinical experience now suggests that gynecologic cancers that at first might have been considered refractory to chemotherapy or to radiation ultimately may be sensitive to SABR (1). With improvements in systemic and biologic chemotherapies that improve efficacy and lower chemotherapy-related adverse events, combined systemic chemotherapy and SABR may serve as a safe therapeutic modality for women with recurrent or persistent gynecologic cancers. Translational clinical trial evaluations of optimally timed and sequenced chemotherapy and SABR are of considerable interest to meet therapeutic needs of women with recurrent or persistent gynecologic cancers.

### Research in context

#### Systematic Review

Our manuscript reports, for the first time, systemic chemotherapy combined with abdominopelvic robotic stereotactic radiosurgery for the treatment of women with recurrent or persistent gynecologic cancers. We searched PubMed with the terms “chemotherapy,” “radiosurgery,” “gynecologic cancer,” and “clinical trial” for publications between January 1, 1999, and March 12, 2015. Only the original single institution robotic stereotactic body radiosurgery phase II trial was found (1). We broadened our publication search and identified three review articles (26–28). These three publications and their referent radiosurgery publications (2–5, 7–14) were selected to frame the context of our phase I trial data.

#### Interpretation

Carboplatin–gemcitabine–SABR treatment in women with recurrent or persistent gynecologic cancer demonstrates safety results that warrant further chemotherapy–SABR evaluations. Chemotherapy–SABR trials combining upfront radiochemotherapy followed by maintenance chemotherapy may be most desirable to lengthen clinical benefit.

## Author Contributions

CK, TS, MM, RE, SL, SW, KZ, KH, and RD treated patients on this trial or contributed to the drafting of this manuscript. This manuscript has been seen, read, and agreed upon in its content by all designated authors.

## Conflict of Interest Statement

The authors declare that the research was conducted in the absence of any commercial or financial relationships that could be construed as a potential conflict of interest.
